# Role of Mast Cells in clearance of *Leishmania* through extracellular trap formation

**DOI:** 10.1038/s41598-017-12753-1

**Published:** 2017-10-16

**Authors:** Nilofer Naqvi, Kavita Ahuja, Angamuthu Selvapandiyan, Ranadhir Dey, Hira Nakhasi, Niti Puri

**Affiliations:** 1 Cellular and Molecular Immunology Laboratory, School of Life Sciences, Jawaharlal Nehru University, New Delhi, 110067 India; 2 JH-Institute of Molecular Medicine, Jamia Hamdard, New Delhi, 110062 India; 3 Department of Bio and Nano Technology, Guru Jambheshwar University of Science and Technology, Hisar, 125001 Haryana India; 4 Division of Emerging and Transfusion Transmitted Diseases, Center for Biologics Evaluation and Research, Food and Drug Administration, Silver Spring, MD 20993 USA

## Abstract

Mast Cells (MCs) are one of the first immune cells encountered by invading pathogens. Their presence in large numbers in the superficial dermis, where *Leishmania* is encountered, suggests that they may play a critical role in immune responses to *Leishmania*. In this study the interactions of *Leishmania donovani*, the causative agent of visceral Leishmaniasis, and *Leishmania tropica*, the causative agent of cutaneous Leishmaniasis with MCs were studied. Co-culture of *Leishmania* with Peritoneal Mast Cells (PMCs) from BALB/c mice and Rat Basophilic Leukaemia (RBL-2H3) MCs led to significant killing of *L. tropica* and to a lesser extent of *L. donovani*. Also, while there was significant uptake of *L. tropica* by MCs, *L. donovani* was not phagocytosed. There was significant generation of Reactive Oxygen Species (ROS) by MCs on co-culture with these species of *Leishmania* which may contribute to their clearance. Interactions of MCs with *Leishmania* led to generation of MC extracellular traps comprising of DNA, histones and tryptase probably to ensnare these pathogens. These results clearly establish that MCs may contribute to host defences to *Leishmania* in a differential manner, by actively taking up these pathogens, and also by mounting effector responses for their clearance by extracellular means.

## Introduction

Mast Cells (MCs) are specialized secretory cells of hematopoietic origin that play a role in innate and adaptive defence to pathogens and in various inflammatory responses. They are found in large numbers in skin, and mucosal lining, and may represent one of the first immune cells encountered by invading pathogens^1^. MCs have been suggested to contribute to protective immunity against bacterial, nonbacterial, and even parasitic pathogens^2^. However, few studies have attempted to provide direct evidence for MC-dependent protective effects in settings of parasite infections that originate in the skin. Thus, the role of MCs as players in anti-parasitic immunity remains to be characterized in detail.

Parasites of the genus *Leishmania*, which are endemic in tropical and subtropical regions around the world, cause a spectrum of diseases ranging from self-healing ulcers to disseminated and often fatal infections, depending on the species involved and on the host immune response. Clinical manifestations of Leishmaniasis include cutaneous Leishmaniasis (CL), muco-cutaneous Leishmaniasis (MCL), visceral Leishmaniasis (VL) and post-kala-azar dermal Leishmaniasis (PKDL). *Leishmania donovani*, the species that causes VL, disseminates to spleen, liver and bone marrow (BM), whereas *L. major* and *L. tropica* that cause CL, remain in the cutaneous lesion and the draining lymph node. 12 million people are affected worldwide by Leishmaniasis and in the Indian subcontinent, about 200 million people are estimated to be at risk of developing VL; this region harbours an estimated 67% of the global VL disease burden^3,4,5^. So far no vaccine and also very limited therapeutic avenues are available to control Leishmaniasis.

In recent years, a lot of research effort has focused on interaction of the innate immune system with *Leishmania* to enable development of better strategies for vaccine and drug development against Leishmaniasis. These studies of host-pathogen interactions have revealed that *Leishmania* parasites have adapted to hide within different cell types to evade the host immune response. Predominantly myeloid cells harbour this obligatory intracellular parasite^6^. Macrophages are crucial for parasite survival, as well as for its elimination^7^. One model called for a ‘Trojan horse’ function of neutrophils whereby apoptotic neutrophils carrying *Leishmania* parasites are transferred to macrophages^8^.

A large numbers of MCs are found in the skin, predominantly in the superficial dermis^9^, where *Leishmania* is encountered after the bite of infected sand flies and their role in *Leishmania major* and *L. donovani* mediated immune responses has been shown. Specifically, *L. major* has been described to activate MCs to induce the release of proinflammatory mediators, and to be phagocytosed by MCs. There is also evidence of their role in establishment of a Th2 response during *L. major* infection^10^. Finally, it has been shown that dermal MCs are required for the recruitment of macrophages during cutaneous granuloma formation, a hallmark feature of parasite induced inflammatory responses^11^. In contrast, substantial inhibition of IL-10, IL-4, and IL-13 expression and the absence of degranulated MCs within the ears of mice immunized with *L. donovani* centrin gene deleted live attenuated vaccine candidate and challenged with virulent wild type parasites, suggested a controlled anti-inflammatory response^12^. As thus observed a distinct dichotomy of opinions exists as to whether MCs can either help or not in the pathogen replication. We, therefore, set out to dissect through this study, the very initial events when MCs encounter *Leishmania* parasites in the *in vitro*. It is important to investigate the role of MCs in Leishmaniasis as: firstly, MCs are markedly more numerous at skin regions that are often infected by *Leishmania* spp.^9^. Secondly, MCs are critically involved in the induction of innate immune responses; and previous studies have suggested that MCs are also involved in the regulation of immunity against various *Leishmania* spp.^10,12,13^. So, the aim of the present study was to determine if MCs are involved in direct clearance of *L. donovani*, and *L. tropica* [the two *Leishmania spp*. that cause very different disease outcomes] promastigotes, by intracellular or extracellular mechanisms, and also to compare the response of MCs to these two *Leishmania spp*. The differential responses of MCs to the two Leishmania spp. may have some bearing on their disease outcomes.

## Results

### Co-culture with MCs reduces the recovery and viability of *L. donovani* and *L. tropica* (promastigotes) *in vitro*

**Figure 1:**
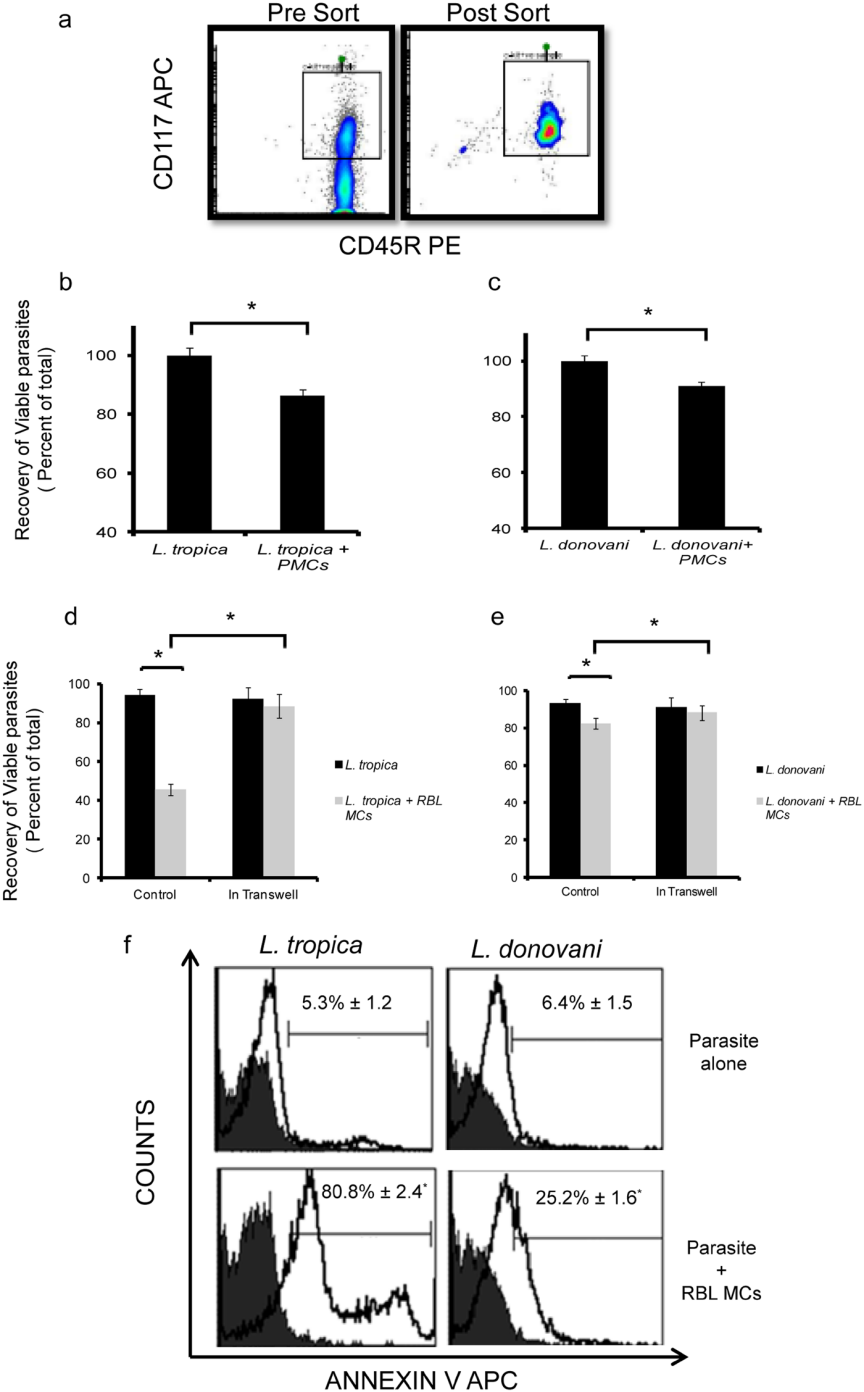
**Cell death of promastigotes on co-culture with MCs.** PMCs were isolated from Peritoneal lavage of female BALB/c mice and were sorted through flowcytometer (Fig. 1a). PMCs were co-cultured with *L. tropica* and *L. donovani* in 96 well plate for 24 h at MOI (1:10). MTT assay was done. The Y-axis represents the relative amount of viable cells after normalization to the Leishmania control (Fig. 1b and c). 0.1 × 10^6^ MCs were seeded in 48 well cell culture plate and cultured overnight in CO_2_ incubator. *L. donovani* and *L. tropica* were added at MOI 1:10. After indicated time points parasites were removed cell viability was counted using trypan blue exclusion method n = 3. Panel d and e represent % cell viability of *L. tropica* and *L. donovani* on co-culture with MCs as well as % cell viability of *L. tropica* and *L. donovani* on co-culture with MCs in transwell system respectively. 0.1 million cells were seeded in 48 well cell culture plate. *Leishmania* were syringe separated and added at MOI 1:10 for 24 h. Parasites were harvested washed followed by adding 50 μl of Annexin Binding Buffer and were processed as mentioned in materials and methods. Representative histograms (solid black line) in panels show Annexin APC positive cells in comparison to unstained (filled grey)(Fig. 1f). Each point represents mean ± SEM of values obtained from three independent assays.

Since large numbers of MCs are found in the superficial dermis^9^, where *Leishmania* is encountered after the bite of infected sand flies, they may have an important role in Leishmaniasis. To directly explore the interaction of *Leishmania* with MCs, promastigotes of *L. tropica* and *L. donovani* obtained from Indian clinical isolates were coincubated with Peritoneal Mast Cells (PMCs) and RBL MCs at multiplicity of infection (MOI) 1:10 for indicated time points and their recoveries and viability were calculated. For isolation of pure population of PMCs, cells of peritoneal lavage were double stained with CD117 APC and CD45R and through sorter 2% PMCs were isolated (Fig. 1a). These PMCs were co-cultured with *L. tropica* and *L. donovani* for 24 h and viability which was done through MTT 3-(4,5-dimethylthiazol-2-yl)-2,5-diphenyltetrazolium bromide assay has reduced to 86.44 ± 2.5% in case of *L. tropica* whereas viability has reduced to 91 ± 1% in case of *L. donovani* (Fig. 1b and c). This reduction in viability by MTT was significant. After 24 h in culture, the recoveries of viable promastigotes of *L. tropica* and *L. donovani*, when cultured alone were 89.3 ± 1.8% and 93.3 ± 2.6% respectively (Fig. 1d and e). There was a marked decrease in the recovery of viable promastigotes of *L. tropica* (45.7 ± 2.3% recovery) when co cultured with RBL MCs, and a small but significant decrease in the recovery of viable promastigotes of *L. donovani* (82.3 ± 2.5% recovery) in presence of MCs (Fig. 1d and e). Further to confirm that the death of promastigotes is because of direct contact with MCs and not due to some soluble mediator released by MCs, the respective promastigotes and MCs were co-cultured in transwell system and recoveries of viable promastigotes calculated. Recoveries of promastigotes alone in the transwells were similar as before. *L. tropica* was 90.5 ± 2.6% and *L. donovani* was 91.2 ± 2.4%. But recovery of viable promastigotes of *L. tropica* with MCs in the transwell system increased to 81.24 ± 1.2%. The recovery of *L. tropica* has significantly reduced from 89.3 ± 1.8% of parasite alone in transwell system to 81.24 ± 1.2% of the parasite along with MCs in nontranswell system. Also, the recovery of viable promastigotes of *L. donovani* co-cultured with MCs in the transwell system was same as recovery of *L. donovani* alone. Hence, promastigotes from both spp. showed better recoveries when co-cultured with MCs in the transwell system (Fig. 1d and e). As promastigotes of *Leishmania* showed reduced viability on co-culture with MCs for 24 h, Phosphatidylserine (PS) exposure on the external leaflet of the plasma membrane of promastigotes was checked to look for signs of death by apoptosis. After 24 h of coculture with RBL MCs, 80.8 ± 2.4% of promastigotes of *L. tropica*, and 25.2 ± 1.6% of promastigotes of *L. donovani* showed binding to Annexin V, confirming PS externalization (Fig. 1f).

### MCs show significant uptake of *L. tropica* but not *L. donovani in vitro*

**Figure 2:**
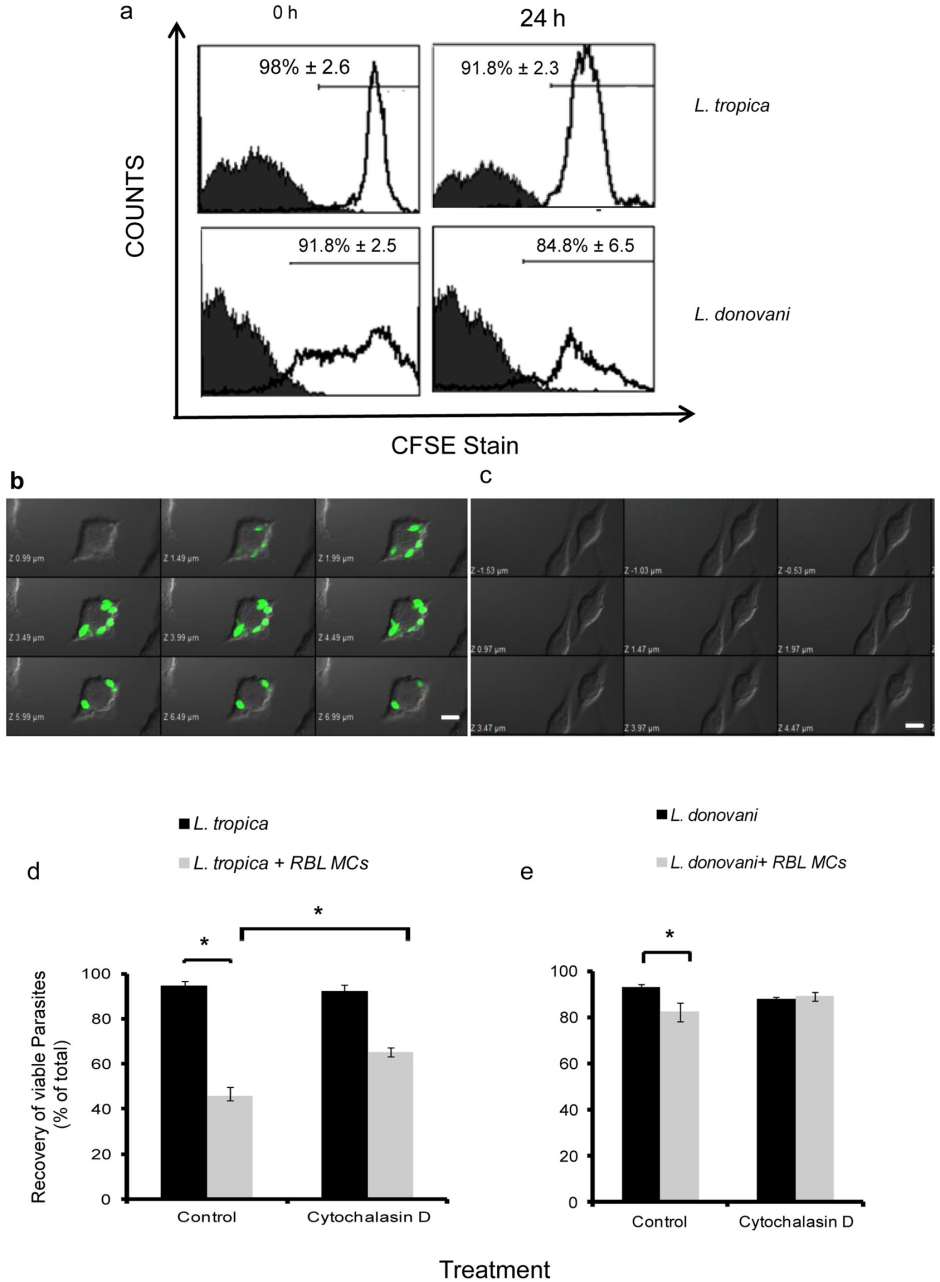
**Uptake of** ***Leishmania*** **by RBL MCs.** Promastigotes were labelled with CFSE and their stability was checked after 24 h as shown in panel a. CFSE labelled promastigotes were syringe separated and incubated with overnight cultured MCs at an MOI 1:10 in 48 well cell culture plate and were processed as discussed in materials and methods. RBL-2H3 were cultured on coverslips overnight. Cells were incubated with CFSE labeled *L. tropica* and *L. donovani* for 24 h and were processed as discussed in materials and methods. Cells were examined using a confocal laser scanning microscope under same settings as shown in Fig. 2b and c for *L. tropica* and *L. donovani* respectively (magnification 100X). Scale bar represents 5 µm. Panel d represents % cell viability of *L. tropica* on co-culture with MCs as well as % cell viability of *L. tropica* on co-culture with MCs treated with Cytochalasin D. Panel e represents co-culture of *L. donovani* on co-culture with MCs as well as % cell viability of *L. donovani* on co-culture with MCs treated with Cytochalasin D. Each point represents mean ± SEM of values obtained from three independent assays.

Since there was an increase in recovery of promastigotes from both *Leishmania spp*. in the transwell system in comparison to non transwell normal co-cultures, and MCs are known to internalise various pathogens^14–17^, the uptake of promastigotes by RBL MCs was studied. To monitor this uptake by flow cytometry or immunofluorescence microscopy, promastigotes of *Leishmania spp*. were labelled with Carboxyfluorescein N-succinimidyl ester (CFSE), and labelling was confirmed by flow cytometry. Promastigotes from both *Leishmania spp*. showed greater than 90% CFSE labelling (Fig. 1a). When these CFSE labelled promastigotes were co-cultured with MCs, 7.2% ± 2.2% and 11.7% ± 2.7% MCs were found to be positive for CFSE labelled *L. tropica* at 18 h and 24 h respectively. But no uptake of CFSE labelled *L. donovani* by MCs was seen at these time points (Table 1). Confocal microscopy also confirmed *L. tropica* being taken up by MCs after 24 h of co-culture (Fig. 2b), and no uptake of CFSE labelled *L. donovani* by MCs at any time point (Fig. 2c). Further to confirm that the reduction in live cell recoveries of promastigotes is because of phagocytosis by MCs, we pre-treated MCs with Cytochalasin D, a known inhibitor of actin polymerization, and therefore phagocytosis^18^, and co-cultured with *L. tropica* and calculated viability. The viability of promastigotes of *L. tropica* at 24 h of co-culture with C﻿ytochalasin D treated MCs was found to be 65.3% ± 4%. Hence the viability still shows a statistically significant decrease from cell viability of promastigotes cultured alone, but a statistically significant increase from viability obtained on co-culture of promastigotes with untreated MCs (Fig. 2d). On the other hand when promastigotes of *L. donovani* were cocultured with Cytochalasin D treated MCs, no change in viable cell recoveries was obtained in comparison to recovery obtained after co-culture with untreated MCs (Fig. 2d). These results again confirm phagocytosis of promastigotes of *L. tropica* but not of *L. donovani* by MCs. Further, since phagocytosis is only partially responsible for reduction in recoveries, other non-phagocytic pathways may also be involved in clearance of *Leishmania* by MCs.

**Table 1:** Uptake of *Leishmania* by Mast Cells

MCs cocultured with the *Leishmania*	Time (h)	*Leishmania* Uptake in MCs (% Gated)	*Leishmania* Uptake in (MFI)
Mast Cells alone	18	1.16 ± 1.2	4.9 ± 2.1
	24	1.8 ± 0.8	4.8 ± 1.6
*L. tropica*	18	7.2 ± 2.2^*^	18.5 ± 2.3^*^
	24	11.7 ± 2.7^*^	23.8 ± 3.4^*^
*L. donovoni*	18	1.9 ± 2.6	5.2 ± 2.3
	24	1.8 ± 3.8	5.1 ± 3.3

0.1 million cells suspended in 1 ml medium were cultured in 48 well plate overnight for adherence and were incubated with late log CFSE labelled *Leishmania* at MOI 1:10 for 18 h and 24 h at 37 °C in CO_2_ incubator followed by harvesting and running through flow cytometer. Each value shown is *Leishmania* uptake in terms of % of total represented as mean ± SEM of values obtained from three ﻿independent assays.

### Generation of Reactive Oxygen Species (ROS) and mediator release from MCs in response to direct interaction with promastigotes of *L. tropica* and *L. donovani*

**Figure 3:**
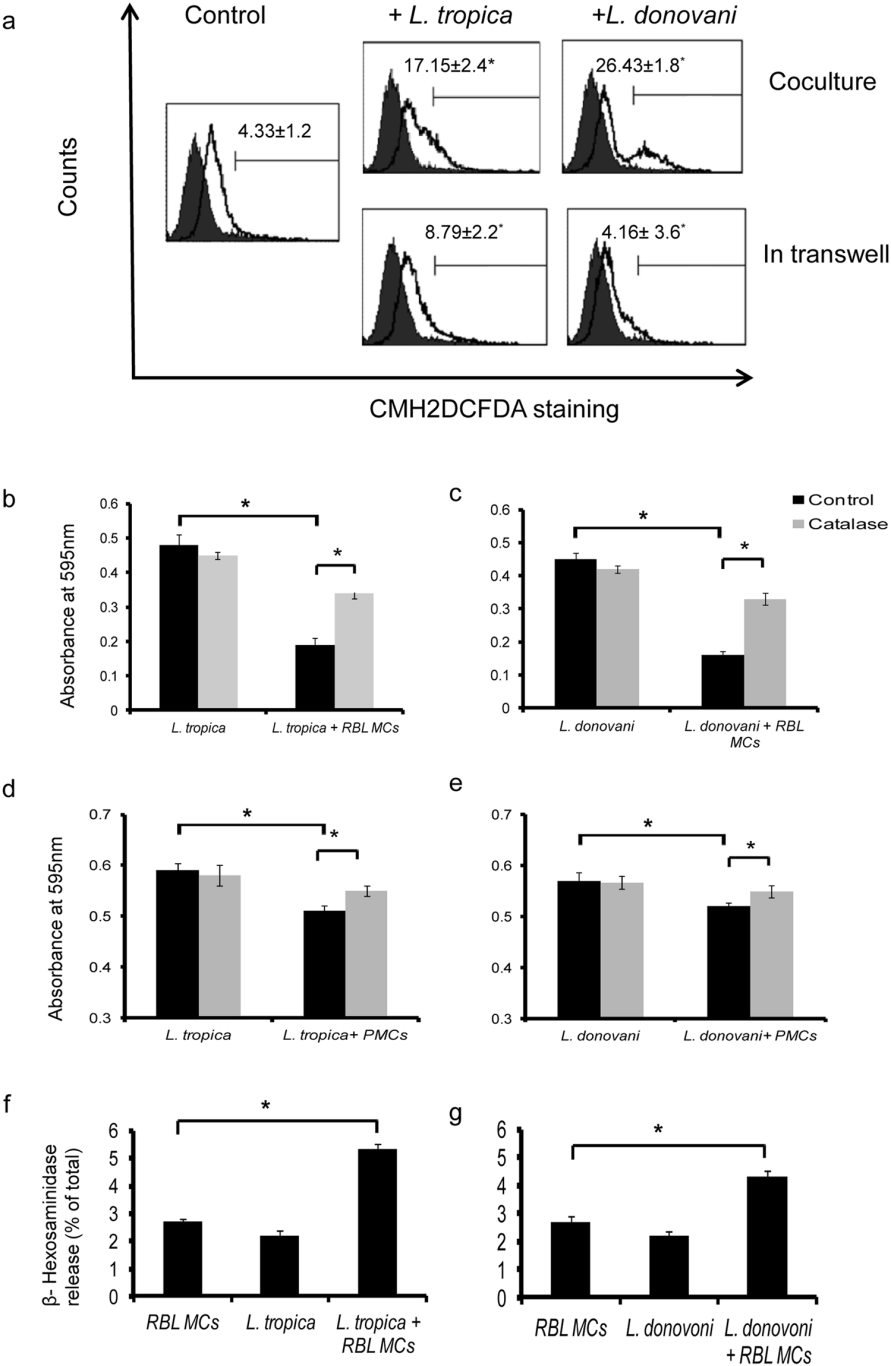
**Generation of ROS and release of mediators by RBL MCs on co-culture of promastigotes.** 0.3 × 10^6^ MCs were seeded in 24 well cell culture plate and *L. donovani* and *L. tropica* were added at MOI 1:10 and were processed as discussed in materials and methods. Representative histograms (solid black line) in panels show CMH2DCFDA stained positive cells in comparison to unstained (filled grey). ROS generation in RBL MCs by *Leishmania* is shown in panel A, n = 3. RBL MCs (1 × 10^4^ cells/well) in 100 µl medium were cultured in a 96-well plate at 37 °C, and co-cultured with promastigotes alone as well as with catalase for 24 h. Cells treated with medium only served as a negative control group. After removing the supernatant of each well and transferring to another 96-well plate 20 µl of MTT solution was then introduced and then processed as discussed in materials and methods. Panel b and c represent effect of catalase on viability of *L. tropica* and *L. donovani*, n = 3. PMCs were cocultured with promastigotes alone as well as with catalase for 24 h. MTT assay was done to assess the viability of promastigotes. Panel d and e represent effect of catalase on viability of *L. tropica* and *L. donovani*, n = 3. 0.1 × 10^6^ cells suspended in 1.0 ml medium were co-cultured with 1:10 MOI *L*. tropica and *L. donovani* for 1 h and were processed as discussed in materials and methods. Panel D and E represent β-hexosaminidase release (% of total) by MCs on co-culture with *L.tropica* and *L. donovani* respectively, n = 5.

There are reports of killing of *Leishmania* by ROS^19–22^. Since we observed cell death in promastigotes we further checked whether ROS are generated in MCs upon co-culture with the promastigotes. ROS generation in control and *Leishmania* co-cultured RBL MCs were estimated by staining MCs with CMH2DCFDA. In untreated controls, 4.33 + 1.2% RBL MCs showed positive ROS generation. We found significantly more ROS generation in MCs upon co-culture with promastigotes of both species compared to their respective controls (Fig. 3a). Co-culture of *L. tropica* at MOI 1:10 after 24 h resulted in 17.15% ± 2.4% cells generating ROS whereas using transwell system ROS generation was 8.79% ± 2.2%. Co-culture of *L. donovani* at MOI 1:10 after 24 h resulted in 26.43% ± 1.8% MCs generating ROS whereas using transwell system ROS generation was 4.16% ± 3.6%. To further determine the functional significance of ROS production catalase was used. Catalase converts hydrogen peroxide into H_2_O and O_2_ and also reduced NET formation in response to PMA activation^23^. So MCs were co-cultured with *L. tropica* and *L. donovani* in 96 well cell culture plate in the presence or absence of catalase. By MTT assay the absorbance at 595 nm of *L. tropica* co-cultured without or with MCs was 0.48 ± 0.03 and 0.12 ± 0.01 respectively as shown in Fig. 3b. Similarly, absorbance at 595 nm of *L. donovani* co-cultured with or without MCs was 0.45 ± 0.02 and 0.23 ± 0.025 respectively (Fig. 3c). Absorbance at 595 nm of *L. tropica* and *L. donovani* when co-cultured with MCs in the presence of catalase was around 0.34 ± 0.015 and 0.33 ± 0.023 respectively (Fig. 3b and c). This increase in OD units in both *L. tropica* and *L. donovani* is statistically significant as shown in Fig. 3b and c. Similar studies were also carried out with PMCs. The absorbance at 595 nm of *L. tropica* co-cultured without or with PMCs was around 0.51 ± 0.01 and 0.59 ± 0.014respectively (Fig. 3d). Similarly, absorbance at 595 nm of *L. donovani* co-cultured with or without PMCs was around 0.52 ± 0.007 and 0.57 ± 0.016 respectively (Fig. 3e). Absorbance at 595 nm of *L. tropica* and *L. donovani* when co-cultured with PMCs in the presence of catalase was around 0.55 ± 0.01 and 0.55 ± 0.012 respectively. This increase in OD units in both *L. tropica* and *L. donovani* is statistically significant (Fig. 3d and e). Since there are also reports of release of MC mediators upon interaction with few species of *Leishmania* ^10^, we checked the release of β-hexosaminidase, as an indicator of MC mediator release on co-culture of both the species. We found low but statistically significant release of β-hexosaminidase by MCs on co-culture with *L. tropica* and *L. donovani* compared to resting MCs alone (Fig. 3f and g).

### Mast cell death by Etosis and release of mast cell extracellular traps (MCETs) on interaction with *L. tropica* and *L. donovani*

**Figure 4:**
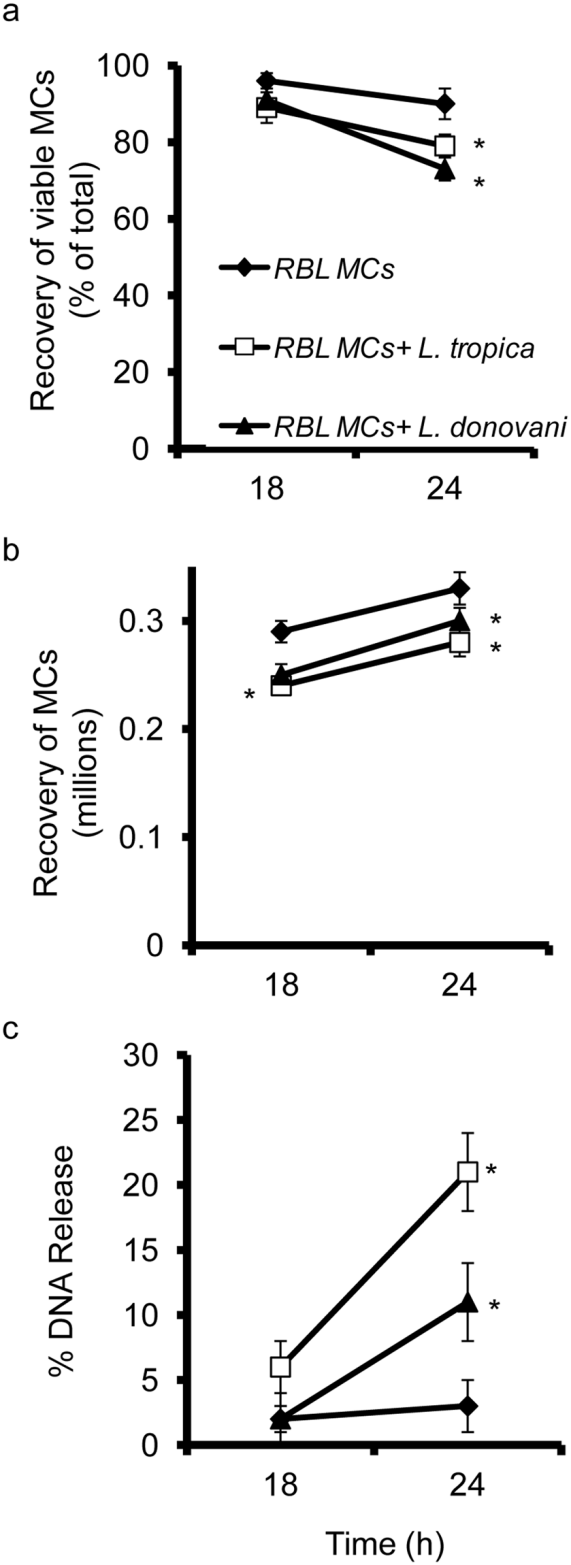
**Cell viability of RBL MCs upon interaction with** ***L. tropica*** **and** ***L. donovani***. 0.1 × 10^6^ MCs were seeded in 48 well cell culture plate and *L. tropica* and *L. donovani* were added at MOI 1:10 and were processed as discussed in materials and methods. Panel a represent recovery of viable MCs (% of total) on coculture with *L. tropica* and *L. donovani*. Panel b represents recovery of live MCs (millions) on coculture with *L. tropica* and *L. donovani*. Panel c represent DNA release in a time and dose-dependent manner as determined with Sytox green. The Y-axis represents the percentage of DNA release after normalization to the mast cell lysis control. Each point represents mean ± SEM of values obtained from three independent assays.

Another mechanism of extracellular killing of pathogens by immune cells is by formation of extracellular traps (ETs)^24^. For extracellular trap formation, MCs are known to die by ETosis and release DNA^15,25–28^. So we checked whether the MCs die on co-culture with promastigotes of these *Leishmania* species. In control RBL MCs the viability was around 96.2% ± 3% at 18 h and 90.5% ± 2.5% at 24 h. The viability of MCs upon co-culture with promastigotes of *L. tropica* fell to 89.5% ± 2.5% at 18 h and 79.3% ± 3.5% at 24 h which is a statistically significant decrease compared to control (Fig. 4a). The viability of MCs upon co-culture with promastigotes of *L. donovani* was also 73.6% ± 5% at 24 h which is a statistically significant decrease compared to control (Fig. 4a). There was a concomitant decrease in the recovery of viable MCs at 24 h of co-culture with promastigotes of *L. tropica* and *L. donovani*, respectively (Fig. 4b). Further, to confirm if MC death was by ETosis, we looked for the release of extracellular DNA by Sytox Green staining after co-culturing MCs with the promastigotes of both species. MCs cultured alone showed very low levels of extracellular DNA (2.3% ± 1.5% at 18 h and 3.2% ± 2.3% at 24 h). Significantly higher release of DNA was seen by MCs on co-culture with *L. tropica* for 18 h (6.5% ± 0.5%), and 24 h (21.6% ± 1.2%) respectively (Fig. 4c), and with *L. donovani* for 24 h (11.7% ± 0.6%).

### Visualization of mast cell extracellular traps containing histones and tryptase in response to *L. tropica* and *L. donovani*

**Figure 5:**
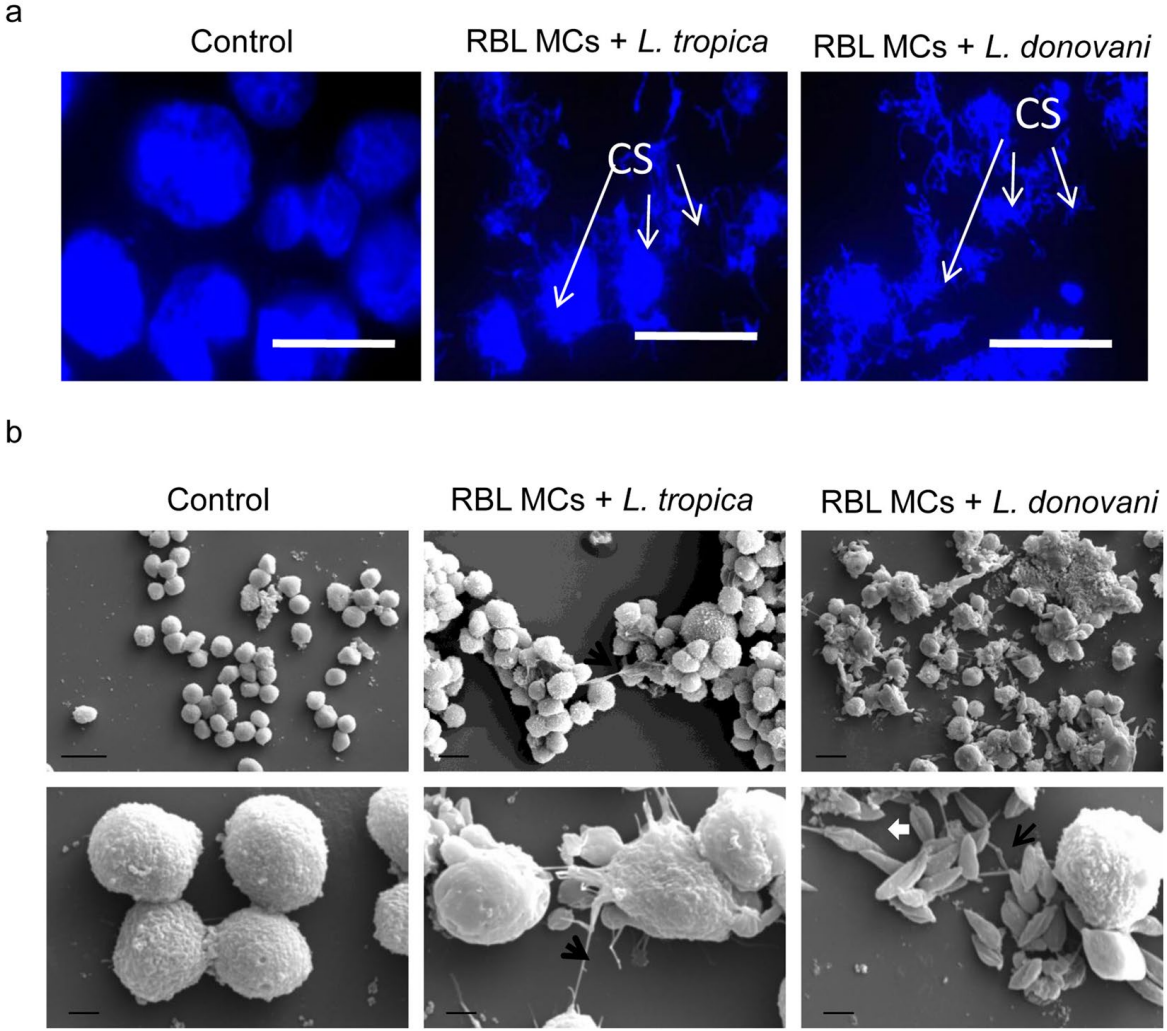
**Visualization of Mast Cell Extracellular Traps.** RBL MCs cultured on a cover glass slip for overnight incubated with promastigotes for 24 h at an MOI of 1:10 in RBL complete medium. Cells were washed with PBS thrice, fixed with methanol mounted in medium having DAPI (blue) and visualised under confocal laser scanning microscope as shown in Fig. 5a. Scale bar is 10 µm and magnification is 100X, n = 3. CS refers to Chromatin Structure. 2.5 × 10^6^ MCs were seeded in 6 well plate for overnight and *L. donovani* and *L. tropica* were added at MOI 1:10. After 24 h parasites were removed cells were harvested and were fixed with 2% glutaraldehyde for 2 h and were processed for Scanning Electron Microscopy. n = 3. Scale bar in top panel of 5b is 10 µm and magnification is 2000X and scale bar in bottom panel of 5b is 2 µm and magnification is 10000X, n = 3. White arrows show *Leishmania* and black arrow could be DNA fibre.

As we saw MCs die by releasing DNA on infection with promastigotes of these *Leishmania* species, we wanted to visualize this phenomenon by microscopy. RBL MCs cultured alone, or co-cultured with promastigotes of *L. tropica* or *L. donovani* were fixed, their DNA stained with DAPI (4, 6-diamidino-2-phenylindole), and visualized by confocal microscope. MCs cultured alone showed DNA staining indicating compact intact chromatin in nuclei, MCs co-cultured with *Leishmania* promastigotes on the other hand showed disrupted/disintegrated chromatin with long extensions as expected in case of MCETs (Fig. 5a). MCs treated similarly were also examined by Scanning Electron Microscopy to confirm release of DNA on infection by promastigotes of these *Leishmania* species. Figure 5b shows no extension formation or release of DNA by non-co-cultured MCs (Fig. 5b, left panel), whereas in MCs co-cultured with *L. donovani* (Fig. 5b, right panel) and *L. tropica* (Fig. 5b, middle panel) we see some extensions of around 0.1 µm diameter. These extensions may be DNA. Images in row 2 are 10000X magnified images and images in row 1 are 2000X magnified images (at least 10 images were visualized in each case). In a few images no *L. tropica* or 1 ± 1 is seen in total 10 images whereas, 20 ± 5 *L. donovani* are seen in total 10 images. Both these microscopic studies reveal the formation of more extensive chromatin extensions or traps by MCs in response to *L. donovani* in comparison to the ones in response to *L. tropica*.

**Figure 6:**
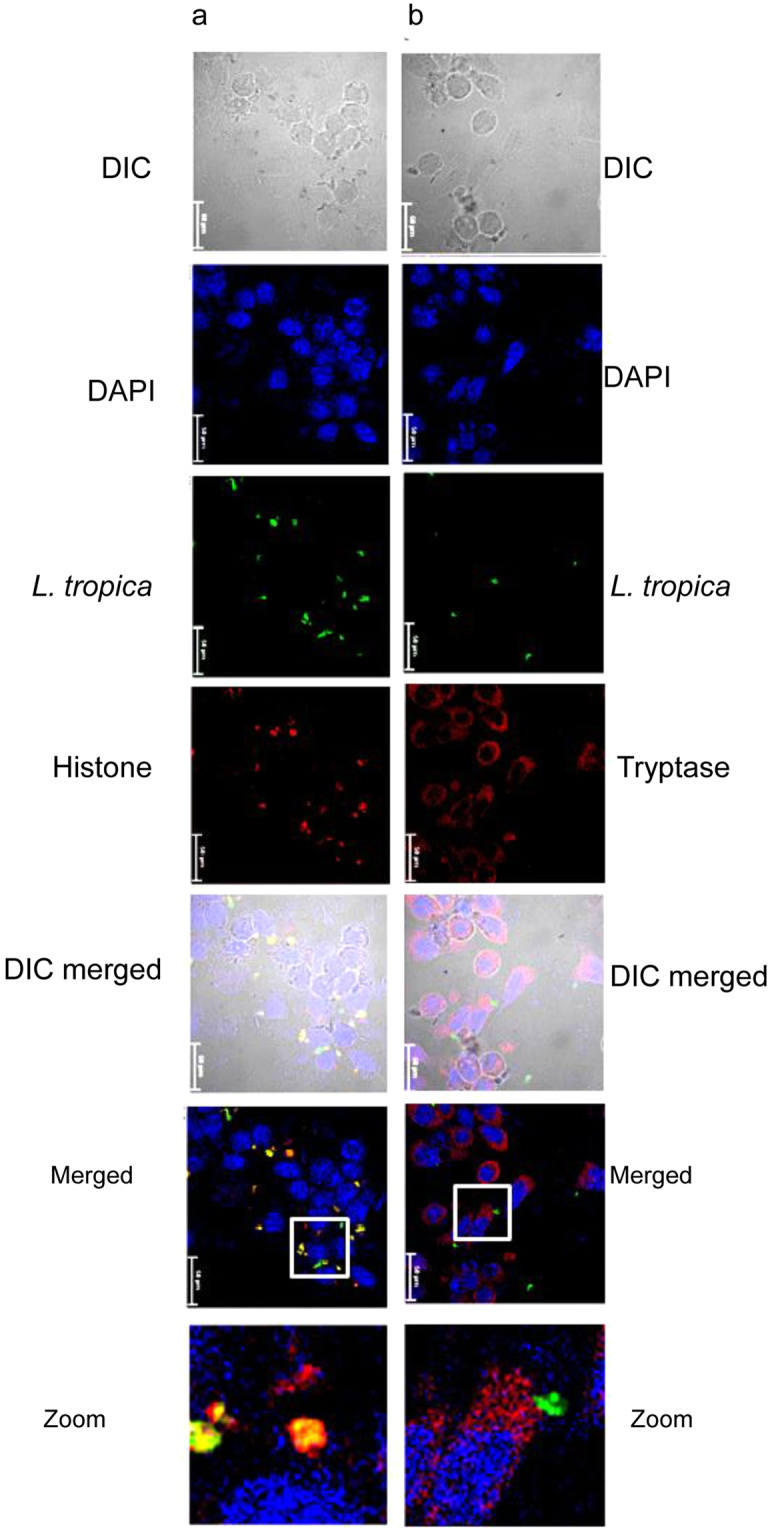
**Immunostaining of MCETs with histone and tryptase on** ***in vitro*** **intreraction of RBL MCs with** ***L. tropica***. Cells cultured on a cover glass slip for overnight incubated with *L. tropica* (green) for 24 h at an MOI of 1:10 in RBL complete medium and were processed as discussed in materials and methods. Panel a represent staining with anti – histone and panel b represent staining with anti – tryptase and were visualized under confocal microscope at 60X. Scale bar is 50 μm, n = 3.

**Figure 7:**
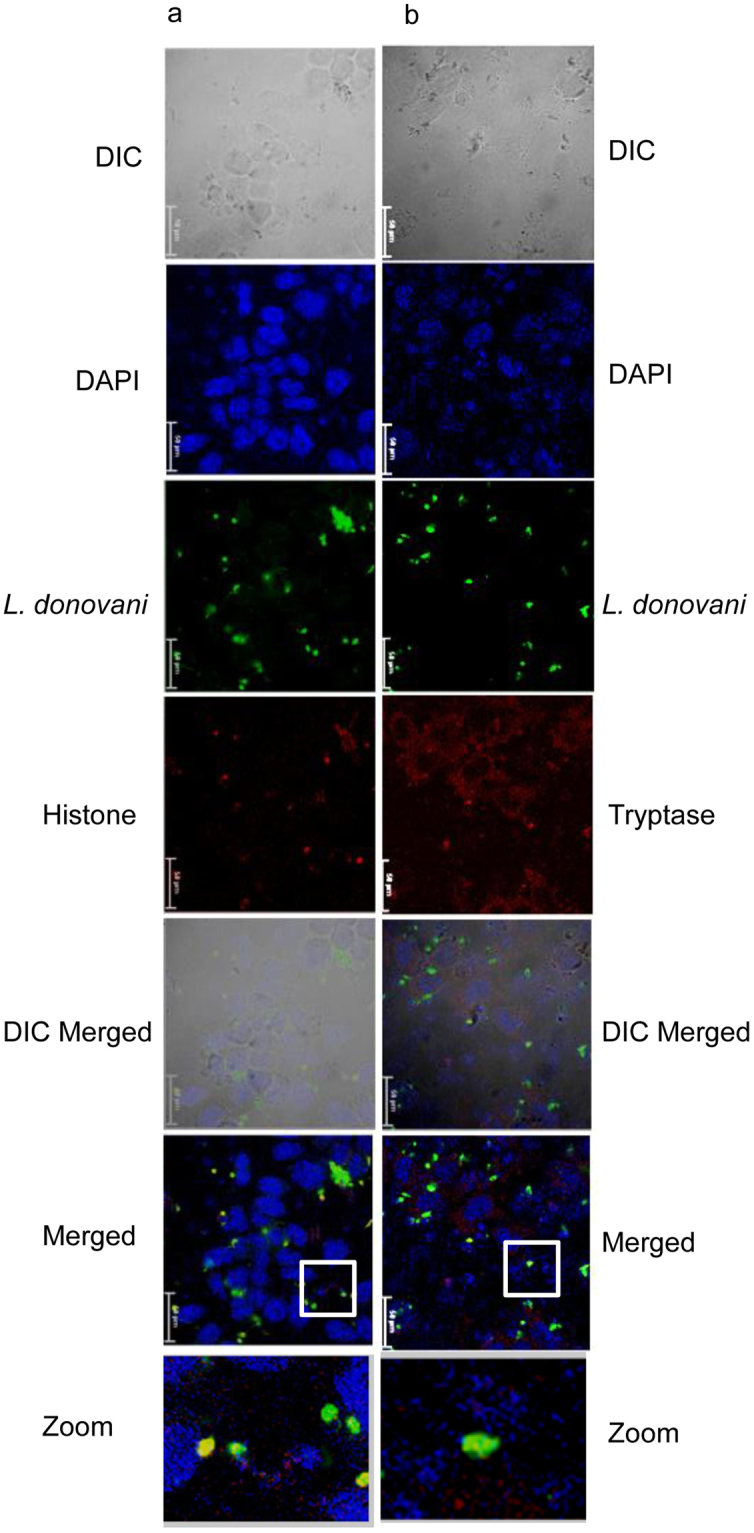
**Immunostaining of MCETs with histone and tryptase on** ***in vitro*** **interaction of RBL MCs with** ***L. donovani***. Cells cultured on a cover glass slip for overnight incubated with *L. donovani* (green) for 24 h at an MOI of 1:10 in RBL medium and were processed as discussed in materials and methods. Panel a represent staining with anti – histone and panel b represent staining with anti – tryptase and were visualized under confocal microscopre at 60X. Scale bar is 50 μm, n = 3.

MCETs comprise of DNA along with histones and tryptase^15,25–28^. RBL MCs were seeded on cover slides and co-cultured with CFSE labelled promastigotes of *L. tropica* and *L. donovani* for 24 h, thereafter DNA was visualized by DAPI staining, tryptase and histones by staining with fluorescently tagged specific antibodies. As is seen in Figs 6 and 7, blue coloured DAPI stained DNA with red coloured tryptase or histone are visible in the extracellular regions. Tryptase staining is extensive in cytosol region of MCs. The images in the insets are further zoomed which depicts co-localized histone (red), DNA (blue) and *L. tropica* (green) in the extracellular regions (Fig. 6a). Similar staining patterns are also seen in the extracellular areas in case of co-culture of MCs with promastigotes of *L. donovani* (Fig. 7). Thus, fluorescence images depict release of tryptase (red), histone (red), and DNA (blue) with extracellular trapped promastigotes of *L. tropica* (green) and *L. donovani* (green) (Figs 6 and 7). The number of promastigotes of *L. donovani* in the extracellular regions are much more than the number of promastigotes of *L. tropica* entrapped in MCETs in all the fields observed.

### Evidence of extracellular killing of *L. tropica* and *L. donovani* by Mast Cell Extracellular Traps

**Figure 8:**
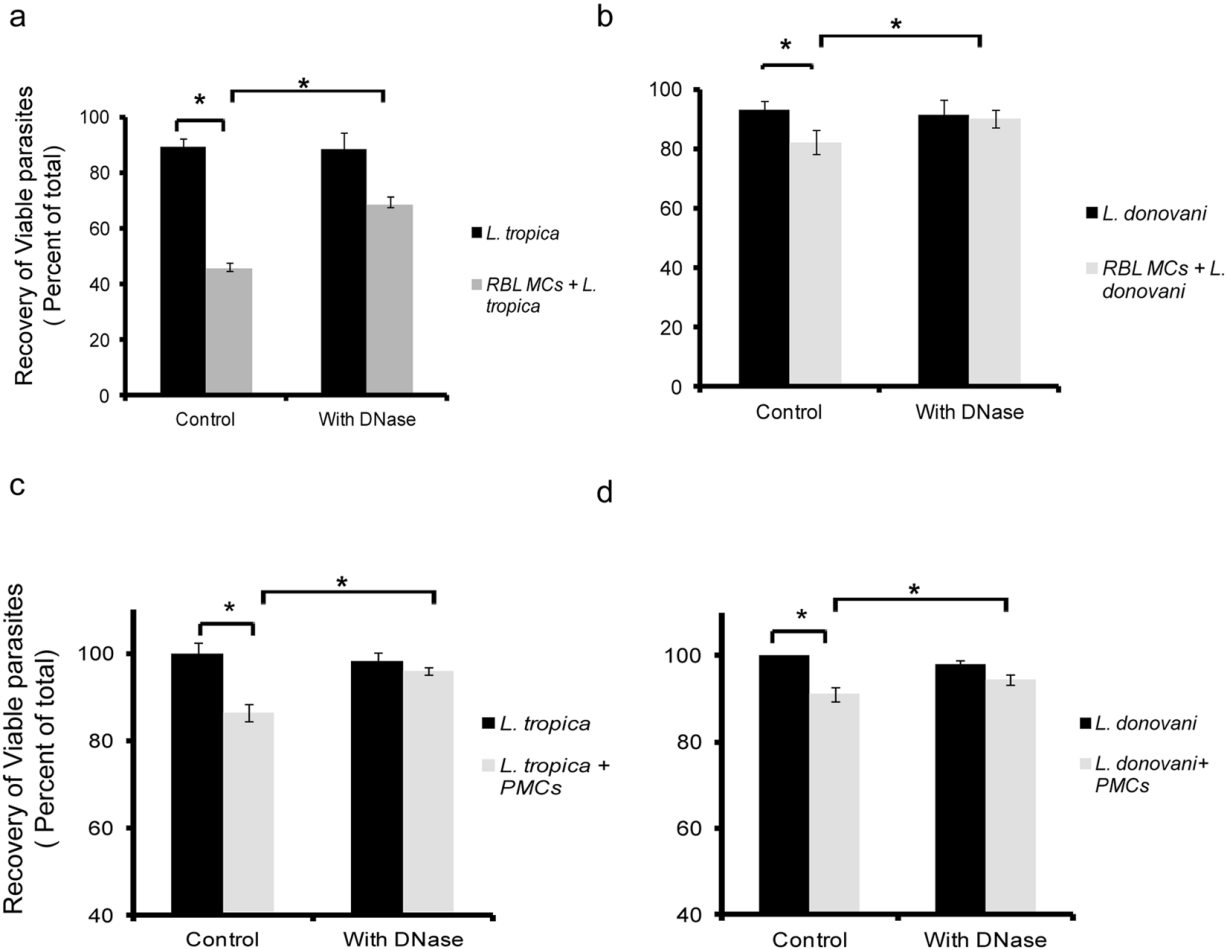
**Evidence of extracellular killing of** ***L. tropica*** **and** ***L. donovani*** **by Mast Cell Extracellular Traps.** 0.1 × 10^6^ MCs were seeded in 48 well cell culture plate and cultured overnight in CO_2_ incubator. Panel 8a and 8b represent recovery of RBL MCs (% of total) by trypan blue on co-culture with *L*. *tropica* and *L. donovani* pretreated with DNase. Panel 8c and 8d represent recovery of PMCs (% of total) by MTT assay co-culture with *L*. *tropica* and *L. donovani* pretreated with DNase. Each point represents mean ± SEM of values obtained from three independent assays.

The results so far indicated release of DNA with histones and tryptase forming MCETs in response to promastigotes of *L. tropica* and *L. donovani*. Further to confirm that the MCETs had a role in killing of these promastigotes it was necessary to check whether disrupting DNA could affect the viability of promastigotes. The cells in co-culture were treated with DNase and the viability of promastigotes of *L. tropica* and *L. donovani* showed a significant increase from 45.7% ± 2.3% to 66.7% ± 4.4%, and 82.43% ± 1.2% to 90.16% ± 2.4%, respectively (Fig. 8a and b). So we conclude that MCET formation is important for killing of promastigotes of *L. tropica* and *L. donovani*.Similarly, when PMCs were co-cultured with *L. tropica* and *L. donovani* for 24 h viability by MTT assay was reduced to 86.44 ± 2.5% in case of *L. tropica* whereas viability was reduced to 91 ± 1% in case of *L. donovani* as shown in Figs 8c and 1d. Further, to confirm the similar effector mechanisms are operating in PMCs as well, DNase treatment was carried out during PMC *Leishmania* co-culture. The viability by MTT assay has significantly increased from 86.44 ± 2.5% to 96 ± 0.8% in case of *L. tropica* and the viability has also increased significantly from 91 ± 1% to 94 ± 1.2% in case of *L. donovani* (Fig. 8c and d). So we conclude that MCET formation is important for killing of promastigotes of *L. tropica* and *L. donovani*.

## Discussion

The initial interactions between a pathogen and host set the stage for the development of long-term immunity. Among the earliest interactions of a pathogen with the immune system will be those involving MCs as they are part of the resident cells at the interface of the host and its environment^1^. The vital role of MCs in triggering the innate immune response has been demonstrated following infections with many bacterial and nonbacterial pathogens, especially intestinal parasites^2^. Only one study so far has looked at the direct interaction between *Leishmania* promastigotes and MCs, and also the specific MC effector responses^10^. Specific genetic deletion of mast cells *in vivo* had different impact on the course of infection with various species of Leishmania^9,29^. There are reports stating *L. major*-infected MC-deficient Kit^(W)^/Kit^(W-v)^ mice developed larger skin lesions than did normal Kit^+/+^ mice^9^ whereas C57BL/6 *Cpa* ^*Cre*^ or BALB/c *Cpa* ^*Ce*^ had no effect when infected with *L. major* ^29^. Others have focused more on recruitment of other cells and inflammatory responses in MC deficient mice^2^. As the role of MCs in *Leishmania* infection is not clear, *we* therefore, explored the very initial events when MCs encounter *Leishmania* parasites in the *in vitro* conditions.

When *L. tropica* and *L. donovani* were co-cultured with PMCs of BALB/c mice, a strain susceptible to leishmaniasis^30^, a reduction in their viabilities was observed. Similar results were obtained on their co-culture with RBL-2H3, a rodent mast cell line that has been extensively used to study MC biology in general and MC-pathogen interactions in particular^17,31–34^. There are reports suggesting role of MCs in infection with *L. major* and *L. donovani* in rats^35,36^. We therefore decided to study the direct interaction of RBL MCs with *L. tropica* and *L. donovani*, as an *in vitro* model to study interaction as would happen in skin just after sand fly bite. Significant decrease was seen in viability of both strains more specifically *L. tropica*, on co-culture with MCs. By using transwell during co-culture studies, we found significant increase in viability of *L. tropica*, as well as *L. donovani*. This indicated that direct cell-cell contact between MCs, and promastigotes of both *Leishmania* species was required for the observed killing of *Leishmania* promastigotes. Compared to *L. tropica, L. donovani* is more viable on co-culture with MCs in 24 h. Moreover, the recovery of *L. tropica* was significantly reduced even in transwell system with MCs. This reduction may be due to some soluble mediators. Previous knockout studies with mice have shown that TLR1, TLR2, TLR3, TLR4 and TLR9 may be important for cytokine response, and other effects in response to *Leishmania* infection^37–39^. Also, in macrophages, the complement receptors, fibronectin receptor, and the mannose-fucose receptor (MR) on the surface of macrophages play important roles in promastigote attachment^40^. Since all these receptors are expressed on MCs as well, they may be involved in direct interaction, and signalling involved in MC response to *Leishmania*.

Killing of *Leishmania* promastigotes by MCs can either be by phagocytosis or by extracellular means by mediator release or Etosis. MCs have been shown to internalise pathogens causing reduction in viability which was dependent on ROS^41^. By our flow cytometric, and microscopic studies with CFSE-labelled promastigotes, a significant phagocytosis of *L. tropica* but not of *L. donovani* by MCs was observed. Cytochalasin D treatment of MCs before co-culture caused a significant increase in cell viability of *L. tropica* in presence of MCs. This validates, that reduction in viable number of promastigotes of *L. tropica* may partially be through phagocytosis by MCs. A previous study has reported that Bone Marrow Derived Mast Cells (BMMCs) can bind and internalise promastigotes of *L. major* and *L. infantum* ^10^. Once inside the MCs, the promastigotes may be killed by ROS generated inside host MCs. We did observe significant ROS production in co-cultured MCs, and also apoptosis of extracellular *Leishmania* parasites, confirmed by Annexin V binding assay. ROS from macrophages has been shown to be required for killing of promastigotes of *L. amazonensis* and regulation of inflammatory responses^42,43^. ROS has also been shown to precipitate apoptosis of the *L. donovani* parasites^44^. Treatment of catalase increased viability of *L.tropica* and *L. donovani* in co-culture studies with PMCs as well as RBL-2H3. As use of transwell system greatly reduced ROS production, we can conclude that direct interaction between cells is required and there does not seem to be much contribution of exosomes from Leishmania to this process.

One interesting observation of our study is that MCs are unable to phagocytose promastigotes of *L. donovani*, but still co-culture with these promastigotes leads to ROS production in MCs. Previous study with neutrophils also report that uptake of *L. donovani* by neutrophils was an infrequent event^45^. Since MCs are not able to take up *L. donovani* and their viability was unaffected by Cytochalasin D treatment of MCs we can rule out the intracellular killing mechanisms. We saw generation of significant amounts of ROS and release of β-hexosaminidase indicating degranulation. This can also be the reason of reduction in viability of *L. donovani*. We have observed release of β-hexosaminidase during initial hour of interaction with *L. tropica* and *L. donovani*. There are earlier reports also which depict that infection with live, virulent promastigotes leads to the release of preformed β-hexosaminidase and TNF-α and *de novo* synthesis of TNF-α in BMMCs^10^.

There are reports of extracellular killing of pathogens by MCs by Etosis^28^. In our study, the interaction of MCs with both *Leishmania* species led to significant death of MCs and release of DNA as confirmed by fluorescence microscopy, electron microscopy, and Sytox green staining. Thus, we found the formation of MCETs in response to both *L. tropica* and *L. donovani*. We found reduction in killing of promastigotes of these parasites by DNase treatment on co-culture with PMCs as well as RBL-2H3. Viability of *L. tropica* increases upto only ~66% on co-culture with MCs along with DNase treatment, which is far less than ~89% when cultured alone, this can be because of phagocytosis or release of toxic antigens of ETosis mainly ROS after digesting the DNA lump, which adversely affects *L. tropica* survival^46^. This leads to the conclusion that promastigotes of both species are susceptible to MCETs. Thus MCETs may not only physically restrict them, but also kill them to some extent. Thus, this is the first report showing direct interaction of MCs with either *L. donovani* or *L. tropica* by direct contact leading to generation of ROS and MCETs. ROS is required to initiate Neutrophil Extracellular Traps (NETs)^46,47^, and similar mechanism may also be operational in MCs. Although *L. donovani* is not phagocytosed by MCs but still causes ROS generation, which may be important for releasing MCETS. In MCs it is possible that MC-specific tryptase and histones, which were both shown to be present in extracellular regions, co-localizing with extracellular DNA and trapped promastigotes by our confocal fluorescence microscopic study, which have also been shown as a component of MCETs previously may have similar antimicrobial functions^32^.

Numerous factors influence disease severity, but the most important determinant of the form of Leishmaniasis is the species of *Leishmania* involved. During this study we were also able to compare the interactions of *L. tropica* and *L. donovani* with MCs and found that both show a different response*. L. tropica* shows greater cell death on interaction with MCs, and is susceptible to phagocytosis but *L. donovani* is not. Secondly, although both *Leishmania* species seem susceptible to MCETs, as shown by reduction in cell death by DNase treatment, but in all our images of MCETs relatively higher number of promastigotes of *L. donovani* in comparison to those of *L. tropica* are seen. These differences could be due to uptake of a proportion of promastigotes of *L. tropica* by MCs, and in addition, may be *L. tropica* promastigotes are more susceptible to killing by MCETs, in comparison to those of *L. donovani*. Previous reports suggest that viability of *L. donovani* is less affected to NETs due to an abundant surface virulence glycolipid lipophosphoglycan (LPG)^48^. *L. donovani* is able to evade killing which is further reconfirming the previously published report^45^. There is a report stating that promastigotes of *L. infantum* induce NET release and evade NET-mediated killing through their 3′- nucleotidase/nuclease activity^49^ and *L. donovani* also constitutively secretes a nuclease which may help in immune evasion^50,51^. Earlier studies also suggest that *L. donovani* exhibits various evasion strategies including alteration of many signalling pathways in macrophages^52–55^. Both species may lead to differential signalling in different host immune cells, as has been shown for *L. major* and *L. donovani* effects on macrophage gene regulation in the past^56^. Differential signalling or different evasion strategies may lead to different pathogenic outcomes.

In conclusion, since MCs are already present in skin and are one of the first immune cells to encounter *Leishmania* promastigotes, they are the ones that respond early. The formation of MCETs is also seen, and they are able to kill promastigotes to some extent, and definitely able to trap them. So, overall it can be concluded that MCs play a very important direct role in early innate immune response to *L. tropica* and *L. donovani* and it will be important to factor in their interaction and effector responses during *Leishmania* infection for success of any vaccine or therapeutic approach to Leishmaniasis.

## Methods

### Animals

Inbred BALB/c female mice (8 to12 weeks old) were used to isolate Peritoneal Mast Cells (PMCs). Animals were obtained from Jamia Hamdard University, New Delhi, India and were maintained in the animal house facility at Jawaharlal Nehru University (JNU), New Delhi, India under pathogen free conditions in positive pressure air-conditioned units (25 °C, 50% relative humidity) at a 12 h light and dark cycle. Both water and food were provided *ad libitum*. Institutional Animal Ethics Committee (IAEC), JNU (registration no: 19/GO/ReBi/S/99/CPCSEA) approved all experimental protocols (IAEC Code: 13/2013) requiring the use of animals. All experiments were performed under relevant guidelines and regulations.

### Isolation and co-culture of PMCs

PMCs were isolated from peritoneal lavage of female BALB/c mice by double staining with APC anti mouse CD117 (Biolegend, San Diego, CA, USA) and PE anti mouse CD45R (Biolegend, San Diego, CA, USA) and sorting through BD FACS Melody. The isolated PMCs were co-cultured in RPMI media supplemented with 20 ng/ml IL-3 (Peprotech, Rocky Hill, NJ, USA) and Stem Cell Factor (Peprotech, Rocky Hill, NJ, USA)^57^ with *L. tropica* and *L. donovani* at MOI 1:10 in 96 well plate.

### Maintenance of cell line

The Rat Basophilic Leukaemia (RBL-2H3) mast cell line was a kind gift from Dr. Paul Roche, NIH, Bethesda, MD, USA. They were maintained in RBL complete medium containing equal parts of Minimum Essential Medium Eagle with Earle’s salts (Gibco, Life technologies, Grand Island, NY, USA) and Iscove’s Modified Dulbecco’s Medium (Gibco, Life technologies, Grand Island, NY, USA) and supplemented with 25 mM HEPES (Sigma, MO, USA) (N-[2- 100 hydroxyethyl]piperazine-N0-[2-ethanesulfonic acid]), 50 µg/ml gentamicin sulfate, and 20% heat- inactivated Fetal Bovine Serum (FBS) (Gibco, Life technologies, Grand Island, NY, USA) in a humidified atmosphere containing 5% CO_2_ at 37 °C. Cell lines were maintained as adherent cultures and subcultured by trypsinization.

### Maintenance of promastigotes of *Leishmania*

Leishmania donovani 1 S (a cloned line from strain 1 S, WHO designation: MHOM/SD/62/1 S), and *Leishmania tropica* (Wright) Luhe (ATCC 50129^™^) were used in all experiments. The promastigotes were cultured and harvested as described previously^58^. Briefly, the parasites were grown *in vitro* in T25 cm^2^ culture flasks at 26 °C in medium 199 (Sigma, MO, USA) (pH 6.8) having 8 μM 6-Biotin (Sigma, MO, USA), 25 mM Hepes, 0.1 mM adenine (Sigma, MO, USA) in 25 mM Hepes), 8 μM hemin (Sigma, MO, USA) [4 mM stock made in 50% triethanolamine (Sigma, MO, USA)], 100 U/ml each of penicillin G (Gibco, Life technologies, Grand Island, NY, USA) and streptomycin (Gibco, Life technologies, Grand Island, NY, USA) and 10% (v/v) heat-inactivated fetal bovine serum.

### CFSE labelling of *Leishmania*

1 million parasites in late log phase were labelled with 5 μM CFSE (Sigma, MO, USA) for 10 min in water bath at 37 °C. After two washes with Phosphate Buffer Saline (PBS) at 2000g for 5 min at 4 °C parasites were syringe separated with 26 ½ gauge needle. Flow cytometric analysis by a BD FACS Calibur flow cytometer at FL1 channel using Cell Quest software indicated that by using this protocol, more than 95% of the *Leishmania* parasites were labelled with CFSE.

### *In vitro* co-culture of MCs and *Leishmania* promastigotes

0.1 million cells suspended in 1 ml medium were cultured in 48 well plate overnight for adherence. They were incubated with late log CFSE labelled *Leishmania* in 1 ml RBL complete medium at MOI 1:10 (parasites counted using haemocytometer) for 6 h ,18 h and 24 h at 37 °C in CO_2_ incubator. MCs were washed 3-4 times to remove the uninfected extracellular parasites. The cells were trypsinized and washed with FACS buffer, fixed in 2% paraformaldehyde (PFA) and analyzed with a BD FACS Calibur flow cytometer in FL1 channel using Cell Quest software. For all FACS experiments, relative fluorescence intensity of 10,000 cells was recorded as single parameter histograms (log scale 1024 channels, 4 decades). Similarly in some co-culture experiments, 0.3 million MCs were seeded in the lower chamber of a transwell polystyrene plate (polycarbonate membrane with 0.4-µm pore size, 6.5-mm diameter; (Corning Costar, Cambridge, MA) and promastigotes were added to the top chamber of the transwell plate in 24 well plate. In some assays, 0.1 million MCs were pre-treated with Cytochalasin D (Sigma-Aldrich) at a final concentration of 10 µg/ml to inhibit phagocytosis and promastigotes were co-cultured. To disrupt MCETs, MCs were pre-treated with 40 U/ml DNaseI (Sigma-Aldrich) to degrade DNA and then promastigotes were co-cultured. To inhibit ROS 25 U/ml PEG catalase (Sigma Aldrich, India) was used. Viability of MCs as well as promastigotes was assessed through trypan blue staining.

### Assessment of apoptosis

MCs were cultured at a concentration of 0.1 million cells/ml in 48 well cell culture plates. After overnight culture, cells were washed with complete medium to remove debris and dead cells. Cells were co-cultured with promastigotes of *Leishmania* for 24 h and promastigotes were removed. Promastigotes were washed and double stained with 7-amino actinomycin- D (7AAD) (Biolegend, San Diego, CA, USA) and annexin V FITC conjugate (Biolegend, San Diego, CA, USA) to assess the apoptotic and necrotic cells using flowcytometry.

### Determination of ROS

MCs were cultured at a concentration of 0.3 million cells/ml in 24 well cell culture plates overnight. Adhered MCs were cocultured with promastigotes directly or in transwell washed with complete medium to remove promastigotes, debris and dead cells. These cells were washed, and resuspended in pre-warmed PBS supplemented with 2% FBS and incubated with 5 μM CMH_2_DCFDA stain (Molecular Probes; Eugene, OR, USA) in the dark for 30 minutes at 37 °C in 5% CO_2_ incubator﻿. The oxidative conversion of CMH_2_DCFDA to its fluorescent product by ROS was measured immediately by BD FACS Calibur flow cytometer in FL1 channel using Cell Quest software.

### MTT assay

To determine cell viability the colorimetric MTT metabolic activity assay was used. MCs in 100 µl medium were cultured in a 96-well plate at 37 °C, and co-cultured with promastigotes for 24 h. Cells treated with medium only served as a negative control group. After removing the supernatant of each well and transferring to another 96-well plate 20 µl of MTT solution (Sigma, MO, USA) (5 mg/ml in PBS) were then introduced. After incubation for another 4 h, the resultant formazan crystals were dissolved in dimethyl sulfoxide (100 µl) and the absorbance intensity was measured by a microplate reader (Spectramax M2, USA) at 595 nm. The relative cell viability (%) was expressed as a percentage relative to the untreated control cells.

### Scanning Electron Microscopy

2.5 × 10^6^ MCs were seeded in 6 well plate for overnight. *L. donovani* and *L. tropica* at their stationary stage from culture were syringe separated and were added at MOI 1:10. After 24 h, parasites were removed and the cells were harvested followed by fixing with 2% glutaraldehyde for 1 h and were processed for Scanning Electron Microscope Zeiss EV040 in Advanced Instrumentation Research Facility.

### Examination of MCETs

RBL cells were seeded on 10 mm diameter coverslips and kept for adherence at 37 °C in CO_2_ incubator. CFSE labelled *L. tropica* and *L. donovani* were added to the cells at MOI 1:10 for 24 h. The medium was removed and cells were fixed with 2% PFA in PBS for 30 min and excess PFA quenched with 50 mM NH_4_Cl in PBS. The PFA fixed cells were then incubated with 3% normal goat serum (Sigma, MO, USA) for 1 h to prevent nonspecific protein binding. Rabbit Anti-Histone H2A (acetyl K5) antibody (Abcam, Cambridge, MA) and Mouse anti-Mast Cell Tryptase antibody (Abcam, Cambridge, MA) diluted in the same buffer were added to the cells, and incubation was conducted for 2 h. After washing, this was followed by 30-min incubation in the presence of secondary goat Abs conjugated to Alexa Fluor 546 (red). Coverslips were mounted in Vecta shield containing DAPI (Vector, Vector Lab. Inc.) to stain nucleus. The images were visualized under Nikon Real Time Laser Scanning Confocal Microscope Model A1R with motorized inverted microscope having Live Cell and Spectral Imaging – Model Ti-E at 60X.

### β-hexosaminidase release

RBL-2H3 cells (0.3 × 10^6^) adhered on plate were washed with RPMI (without phenol red) (Sigma, MO, USA) and co-cultured with *L. donovani* and *L. tropica* in a final volume of 1.5 ml. Plates were incubated at 37 °C, aliquots of the medium were withdrawn at various times and β-hexosaminidase activity released into the medium was measured. Mock degranulation studies were carried out in parallel by using medium alone^59^. To determine β-hexosaminidase activity, aliquots of the supernatants and cell lysates were incubated with the substrate solution ((Sigma, MO, USA) 1.3 mg/ml of *p*-nitrophenyl- *N*-acetyl-β-D-glucosaminide in 0.1 M citrate buffer (pH 4.5) for 90 mins at 37 °C. Absorbance was read at 405 nm and the amount of exocytosis was expressed as the percentage of total β-hexosaminidase activity released in the supernatant.

### Quantification of DNA released by MCs

DNA release by MCs was quantified using a Sytox Green-based assay. MCs (5 × 10^4^ cells/well) were seeded in a 96 well plate wrapped in aluminium foil in the presence of 2.5 μM Sytox Green (Molecular Probes; Eugene, OR, USA) and infected with promastigotes of *L. donovani* and *L. tropica* - MOI 1:1, 1:10- or left untreated. The same amount of *L. donovani* and *L. tropica* served as viability control whereas triton-lysed MCs served as a reference for 100% DNA release.

### Statistics

Statistical analysis was performed using Graph Pad Prism 5 software (San Diego, USA). The significance of any difference was calculated by using Mann-Whitney U Test. *p < 0.05 represent statistically significant difference between the samples.
